# Repetitive Transcranial Magnetic Stimulation in Stroke: A Literature Review of the Current Role and Controversies of Neurorehabilitation Through Electromagnetic Pulses

**DOI:** 10.7759/cureus.41714

**Published:** 2023-07-11

**Authors:** Paula Vallejo, Emily Cueva, Pablo Martínez-Lozada, Cecilia A García-Ríos, Diego H Miranda-Barros, Jose E Leon-Rojas

**Affiliations:** 1 Medical School, Universidad de Las Américas, Quito, ECU; 2 Medical Research Department, NeurALL Research Group, Quito, ECU; 3 Pediatrics, Escuela Superior Politécnica de Chimborazo, Riobamba, ECU; 4 Neurological Surgery, Universidad de Las Américas, Quito, ECU; 5 Research and Development Department, Medignosis, Quito, ECU

**Keywords:** tms, repetitive transcranial magnetic stimulation (rtms), rtms, neurorehabilitation, neuronal plasticity, stroke

## Abstract

Repetitive transcranial magnetic stimulation (rTMS) is an effective method used for the treatment of various neurological diseases, including stroke, epilepsy, and movement disorders. The pathophysiological mechanism for the effect of TMS is not clear. In this literature review, we conducted a detailed search regarding the effect of rTMS on neurotransmission and neuronal plasticity through the modulation of neuronal excitability. Evidence suggests that intramolecular subatomic mechanisms, including genetic changes related to neuronal prevention and death, play an important role. We also discuss the use of rTMS in the rehabilitation of patients with stroke and its main complications, as well as alternative mechanisms related to recovery, emphasizing the findings of available evidence and touching on possible controversies and limitations of the method.

## Introduction and background

Transcranial magnetic stimulation (TMS) is a non-invasive method that is based on electromagnetic induction of the brain tissue [1]. Currently, various repetitive TMS (rTMS) protocols are available, which transformed from being an experimental tool to providing a novel therapeutic option to modulate cortical excitability in sensory, cognitive, and motor functions in patients with neuropsychiatric diseases [1,2]. This technique has also been used for stroke rehabilitation for hand motor recovery, post-stroke acute motor impairment, and chronic non-fluent aphasia [1,2]. rTMS is applied through electric current pulses of varied intensity through a condenser placed on the patient’s scalp; these pulses generate a perpendicular magnetic field on the brain tissue. The selective stimulation or inhibition of neurons can induce neuronal plasticity, which depends on the shape, size, type, and orientation of the coil, as well as on the strength and frequency of the magnetic pulses [1].

**Figure 1:**
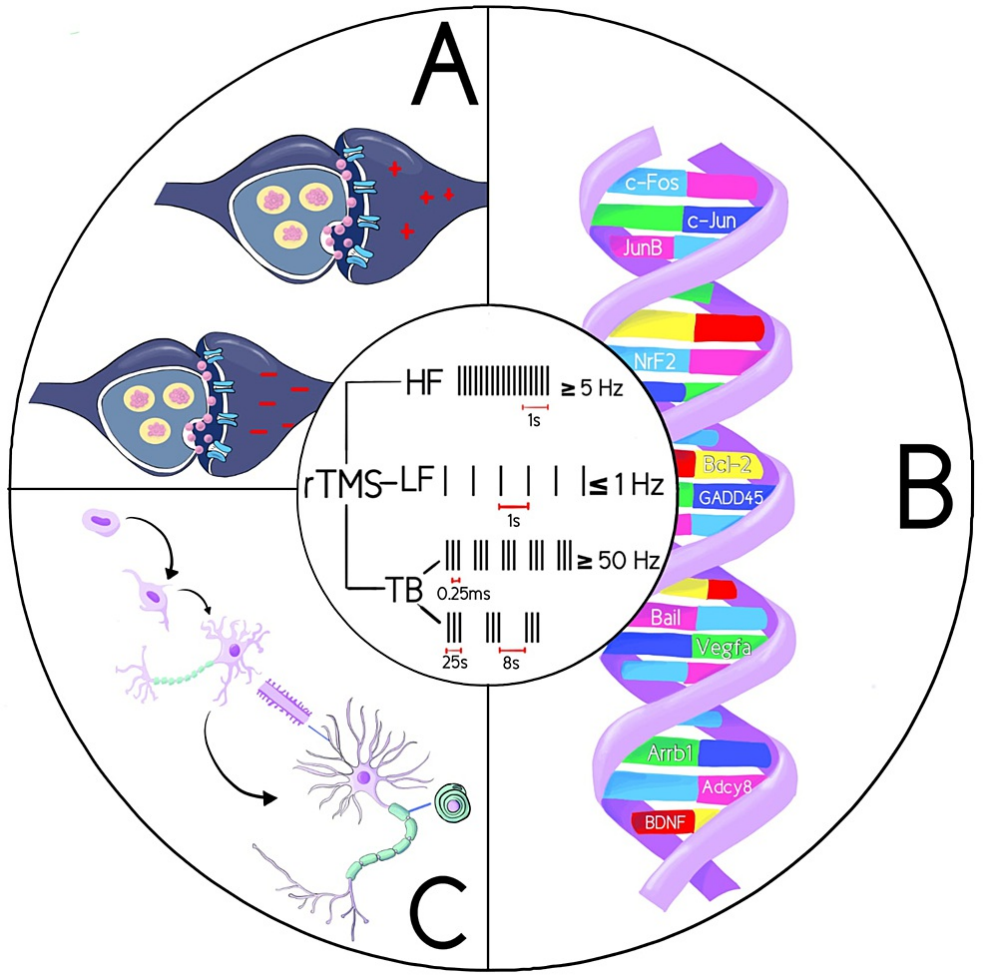
Graphical representation of the biological effects of rTMS. A: rTMS induces changes in synaptic plasticity and conduction, stimulating and inhibiting neuronal connections. B: rTMS stimulates specific gene expression. C: rTMS changes neuronal morphology and can induce neurogenesis. Finally, in the center, we can see a graphical representation of the different types of rTMS based on the frequency of stimulation. Image credits: Emily Cueva. rTMS = repetitive transcranial magnetic stimulation; ADCY8 = adenylyl cyclase type 8; ARRB1 = arrestin, beta 1; Bcl-2 = B-cell lymphoma 2; BDNF = brain-derived neurotrophic factor; GADD45 = growth arrest and DNA-damage-inducible protein 45 alpha; NrF2 = nuclear factor erythroid 2-related factor 2; VEGFA = vascular endothelial growth factor A; HF = high frequency; LF = low frequency; TB = theta bursts

The effect on neuronal excitability is widely influenced by the rate or frequency at which the pulses are sent. The two main types are described as high-frequency rTMS (HF-rTMS) (>5 Hz) and low-frequency rTMS (LF-rTMS) (<1 Hz) [3]. Both protocols are considered safe therapeutic options; however, LF-rTMS is considered safer and superior to HF-rTMS. Studies have reported a higher risk of seizures with frequencies of 20-25 Hz without an increased risk of developing epilepsy [1]. However, the adverse effects of both techniques are usually mild and temporary [3]. The molecular mechanisms of rTMS continue to be investigated, one of which is the ability to modulate gene expression and with it the production of transcription factors [2,4-6]. From a functional viewpoint, rTMS modifies the activity of neurotransmitters and their receptors, which makes it possible to facilitate or inhibit neuronal synaptic connections [2,4-6]. In this way, the processes of neuroplasticity and neuronal reorganization are accelerated. These therapeutic effects are corroborated by the improvement of physical deficits after a stroke. Figure 1 provides a graphical summary of the biological effects of rTMS.

This review presents the neurophysiological mechanisms by which rTMS is believed to work and its therapeutical properties for patients who have survived a stroke. We discuss varied and common physical deficits that arise after stroke, such as motor dysfunction in the upper and lower limbs, spasticity, balance disorders, aphasia, and dysphagia, as well as the role that rTMS might play in their rehabilitation.

## Review

Neurophysiology and plausible mechanisms of repetitive transcranial magnetic stimulation

To fully understand the neurophysiological mechanisms behind rTMS, the inner workings of TMS need to be revised. TMS is delivered through pulses of varying frequencies via a wire coil positioned over the head of the patient that generates rapidly changing perpendicular magnetic fields by short electrical current discharges [6]. The magnetic fields, in turn, induce circular electrical currents within the cerebral cortex in a plane parallel to both the coil and the scalp of the patient [6]. These currents activate cortical pyramidal cells and influence consequent action potentials along the corticospinal tract, inducing motor-neuron firing that can be detected by surface electrodes that record motor-evoked potentials (MEPs) through electromyography of the targeted muscles [4]. The outcome is the selective depolarization of cortical neurons which can significantly impact neuroplasticity, gene expression, and even the survival and genesis of neurons [1,4].

rTMS is a hypernym that includes any TMS protocol that consists of three or more pulses of a particular intensity delivered at 0.5 pulses/second as minimum frequency [7]. rTMS protocols can be categorized into three groups, namely, high frequency, low frequency, and theta burst stimulation [8]. Each protocol differs according to the number and intensity of pulses as well as the frequency at which they are delivered [8]. There is evidence that LF-rTMS protocols consisting of pulses of 1 Hz or less can cause inhibitory effects, while HF-rTMS involves pulses of 5 Hz or more and results in excitatory effects aimed at modifying cortical excitability [9]. In a typical HF or LF-rTMS intervention, identical and individual stimuli are spaced by equal inter-stimulus intervals (ISIs) [6]. Novel theta burst stimulation (TBS) protocols have been described, which are based on the theta rhythm present in human neural oscillatory activity [6]. Bursts of high-frequency pulses (typically three to five 50-100 Hz pulses) at 5 Hz with an ISI of 0.2 seconds are delivered [5,7]. In intermittent TBS protocols (iTBS), repeating cycles of TBS are given for two seconds, followed by a pause of eight seconds [6]. In general, iTBS results in cortical excitation [7]. Furthermore, continuous TBS (cTBS) consists of 40-second TBS stimulation without pause and generally results in cortical inhibition [6,7]. The advantage of rTMS protocols is that the effects on neuronal plasticity are longer lasting compared to other non-invasive stimulatory therapies [2].

Long-term potentiation and long-term depression phenomena

Although the neurophysiological mechanisms have not been elucidated, it is believed that the main mechanism underlying the alteration of neuronal excitability, responsible for the therapeutic effect of rTMS, is related to two phenomena, namely, long-term potentiation (LTP) and long-term depression (LTD) [2,4,6]. As previously mentioned, pulses higher than 5 Hz of high frequency generally facilitate the activation of neural networks or LTP-like effects [4]. Based on the current literature, the proposed mechanism behind the neuronal plasticity induced by LTP of rTMS is the simultaneous depolarization of pre- and post-synaptic neurons [4]. On one side, depolarization of the pre-synaptic neuron results in the release of glutamate into the synapse; simultaneously, depolarization in dendrites of the post-synaptic neuron opens voltage-gated calcium channels and removes the magnesium block from N-methyl-D-aspartate (NMDA) receptors [4]. Together, these events during rTMS therapy stimulate the accumulation of α-amino-3-hydroxy-5-methyl-4-isoxazolepropionic acid (AMPA) receptors in the post-synaptic neurons and may be responsible for synapsis strengthening and LTP-like outcomes [4].

In contrast, pulses of 1 Hz or less of low frequency tend to cause LTD-like effects. However, it is not clear whether rTMS induces LTD across the synapse by mechanisms similar to those mentioned above [4]. If a low-frequency stimulation (<1 Hz) is given at an intensity below the motor threshold (MT), the stimulus will have an inhibitory effect [6]. In addition, the stimulation will suppress an MEP in the targeted muscle only if the muscle is at rest, and if high-frequency rTMS below the MT is given before the low-frequency stimuli, the LTD effects could be intensified. These LTD-like effects could last for 60 minutes [6]. It is important to note that both the stimulation frequency and intensity as well as the number of pulses delivered can affect the time length of stimuli. For example, pulses below the MT are considered low intensity and tend to reduce neuronal activity compared to pulses greater than the MT, which are high intensity and tend to promote neuronal activity [6]. However, these effects are not homogeneous and may be the outcome of targeting and stimulating different neuron populations in the cortex [9]. Klomjai et al. have suggested that there are two phases concerning synaptic changes derived from LTP and LTD [6]. A short or early phase corresponds to changes that last for 30 to 60 minutes only and a long or late phase in which protein changes or synthesis occur [6]. Significant effects during the late phase might be key to observing functional changes in the patient, as changes in gene expression and proteins may modify molecular cascades leading to therapeutic outcomes.

Changes in gene expression and its effects

Protein production is determined by gene expression patterns, and rTMS has been shown to influence its regulation. For instance, several animal studies have found that rTMS can modify the expression of more than 50 genes, where most of them influence key processes such as neuroprotection, neurotransmission, and neuronal plasticity [2,4-6,10,11]. Please refer to Table 1 for a list of relevant genes, their function, and the effect of rTMS. Among the genes related to inflammation and neuronal injury, rTMS influences the expression of several genes, including *Fos*, *Jun*, and *JunB*. In addition, experimental studies that measured the changes in gene expression followed by middle cerebral artery occlusion found that *Fos* and *Jun* were significantly upregulated after iTBS, being the most effective among the different rTMS protocols [5]. These genes are known to play a vital role during the initial response to acute ischemic-reperfusion brain injury [1,5]. Significantly, *c-Fos* and *c-Jun* also control the expression of growth factors such as brain-derived neurotrophic factor (BDNF) [1].

**Table 1:** Genes and proteins influenced by rTMS treatments and their presumed therapeutic function. *: This is not an exhaustive list. rTMS = repetitive transcranial magnetic stimulation; BDNF = brain-derived neurotrophic factor; NrF2 = nuclear factor erythroid 2-related factor 2; CNS = central nervous system; Bcl-2 = B-cell lymphoma 2; BAIL = Bronsted acid ionic liquid; VEGFA = vascular endothelial growth factor A; MAP = microtubule-associated protein; NMDA = N-methyl-D-aspartate; AMPA = α-amino-3-hydroxy-5-methyl-4-isoxazolepropionic acid; GADD45 = growth arrest and DNA-damage-inducible protein 45 alpha

Reference	Gene/protein	Functionality	Effect after rTMS	Presumed therapeutic function
[2,5]	c-Fos	Neuroprotection, neuronal remodeling and repair	Upregulated	Enhanced repair and remodeling
[2,5]	c-Jun	Apoptosis, cellular repair and remodeling	Upregulated	Enhanced repair and remodeling
[1,2,5]	BDNF	Growth factor, neurogenesis, neuronal survival, regeneration, remodeling, plasticity	Upregulated	Promote neuroplasticity, motor learning, dendritic growth, regeneration
[11]	Nrf2	Antioxidant, stress, and defensive responses against inflammatory CNS diseases	Upregulated	Anti-inflammatory effects
[2,12]	Caspase-3	Apoptotic protein	Downregulated	Promote survival
[2]	Bcl-2	Antiapoptotic factor, memory and learning improvement	Upregulated	Promote survival and learning
[5]	BAIL and VEGFA	Angiogenesis	Upregulated	Promote angiogenesis in the lesioned site
[5]	Arrb1, Adcy8	GPCR signaling	Upregulated	Promote neuroprotection and plasticity
[2]	MAP	Cytoskeletal protein	Upregulated	Neuronal function recovery
[2]	NMDA	Neuroplasticity	Upregulated	Promote neuroplasticity
[2,4]	AMPA	Neuroplasticity	Upregulated	Promote neuroplasticity
[5]	Gabbr1	Production of GABA_B _receptor	Upregulated	Promote neuroplasticity
[5]	Gad1, Gad2	GABA biosynthesis, code for GAD65 and GAD67 (glutamate decarboxylase isoforms)	Upregulated	Promote neuroplasticity
[5]	GADD45	Stress response	Upregulated	Support adaptive cell responses

BDNF has been associated with numerous functions such as learning, neuroplasticity, neuronal survival, and increasing the function of surviving neurons [2,5]. Although the mechanism remains unknown, experimental studies in rodents have demonstrated the upregulation of BDNF in the brain, even when the intensity of stimulation is very low. This is further supported by serum tests in humans that have also shown an increase in BDNF after rTMS [4]. In healthy volunteers, serum BDNF concentrations increased after high-frequency stimulation and decreased following low-frequency stimulation [10]. Additionally, Wang et al. have shown that the affinity of BDNF to the tyrosine receptor kinase B (TrkB) is increased, and with this the association between TrkB and the NMDA receptor. This might suggest that this interaction may contribute to synaptic plasticity [10].

rTMS can control the activity of the nuclear factor kappa B (NFKB) associated with neuronal death and nuclear factor erythroid 2 (NF-E2)-related factor 2(Nrf2) responsible for oxidative damage and induce the production of proinflammatory cytokines [1]. Tian et al. studied the effect of rTMS on the expression of Nrf2 in the hippocampus in an experimental model using rats with patterns of depression and anxiety [11]. The authors found that Nrf2 was upregulated after a week of daily rTMS treatment. In addition, they found that inflammatory cytokines decreased and ultimately the anxiety and depressive-like symptoms improved [11]. They suggested that the anti-inflammatory effect of rTMS improves behaviors related to depression and anxiety [11].

Regarding genes associated with angiogenesis, *BAI1 *and *VEGFA *were significantly upregulated after iTBS stimulation [5]. Promoting angiogenesis may have a positive impact on functional recovery after stroke through endogenous mechanisms, such as resolution of edema and necrotic tissue, and reperfusion of ischemic penumbra, which occur in the recovery phase [5]. Furthermore, the protein growth arrest and DNA-damage-inducible protein 45 alpha (GADD45) was found to be significantly upregulated following iTBS [5]. As this protein is involved in regulating cellular responses to stress, enhanced GADD45 expression can favor neurovascular remodeling and repair in the perilesional cortex after injury [5]. In addition, increased G-protein-coupled receptor (GPCR) signaling suggests that synapse formation and neural plasticity are promoted. *Arrb1*, *Adcy8*, and *BDNF *are genes involved in GPCR signaling and appear to be upregulated after rTMS-type protocols [5].

The molecular pathways of apoptosis also have an impact on the spread of ischemic brain injury after stroke. A study in mice found that HF-rTMS downregulated caspase-3 expression, indicating that the therapy inhibited apoptosis, thus reducing brain damage [12]. Similarly, the antiapoptotic factor Bcl-2, which is involved in memory and learning, may increase after rTMS [2,5].

Further molecular processes enhancing rehabilitation

Effects on neuronal survival, neuronal morphology, and neurogenesis may also boost recovery in post-stroke patients, and there is evidence that rTMS can influence these processes [1,2,5]. Studies in rats have shown that chronic administration of a daily session of HF-rTMS (25 Hz) results in increased neurogenesis in the dentate gyrus of the hippocampus [4]. However, further studies are needed to determine the survival and function of these nascent neurons.

Different ischemia animal models have shown that rTMS may have a neuroprotective role, influencing neuronal metabolism and helping in recovering correct functionality in the affected neurons [2]. For example, an experimental study in rats revealed that rTMS increased the ATP content and expression of cytoskeletal protein microtubule-associated protein 2; therefore, rTMS can become an adjunctive therapy in neuronal recovery for cerebrovascular disease [13].

Furthermore, in vitro laboratory studies of rTMS using cells from the hippocampus stimulated with high frequency (10 Hz) showed structural changes in small dendritic spines of CA1 pyramidal neurons [4]. Moreover, 18F-fludeoxyglucose micro-positron emission tomography images in rats found increased glucose metabolism in the cortex and striatum following rTMS compared to sham treatments [12]. All these factors may support endogenous mechanisms to enhance recovery and can help in elucidating the basis of the therapeutic effects of rTMS.

Transcranial magnetic stimulation use after a stroke

In a healthy brain, there is a balance of cortical excitability between the two cerebral hemispheres, but this balance is affected after a stroke. In the affected hemisphere, both excitability and homonymous motor representation decrease, while the non-affected hemisphere has higher excitability [3]. This overexcitability can be reduced with the use of rTMS, as well as by increasing the cortical excitability of the lesioned hemisphere. HF-rTMS stimulates the lesioned hemisphere to increase its excitability while LF-rTMS reduces it [3]. rTMS protocols in rehabilitation have aimed to increase plasticity and improve motor function based on the interhemispheric competence model, that is, to avoid transcallosal inhibition of the affected hemisphere by the unaffected hemisphere [14]. Different randomized studies have shown that rTMS is highly effective in helping the recovery of motor functions in patients who have had a stroke in the acute stage of recovery [15]. For example, in lower limb activities, rTMS-treated groups demonstrated a higher level of improvement than placebo groups in chronic stroke patients [14].

Recovery of motor function

Concerning motor recovery, the compromised motor areas can recover their function through somatotropic reorganization to increase motor learning and performance. The premotor cortex (PM) and the supplementary motor area (SMA) play a relevant role in the generation of neural networks and alternative motor output pathways [16]. As discussed before, neural plasticity is mediated in two ways. The first is LTP which can be defined as long synaptic enchantment, and the second is LTD, defined by the dismissing or decrease of neural activity [17]. To change LDP and LTP there exist different protocols using TMS, such as LF-rTMS or HF-rTMS [16]. Moreover, when low-frequency TMS is used, motor rehabilitation is increased by decreasing the amplitude of MEP in the counter-injured primary motor cortex and the duration of inhibition of the transcallosal pathway. On the other hand, when HF-rTMS is applied with neurorehabilitation in the primary motor cortex of the injured hemisphere, improvement in motor symptoms is evident [1]. Additionally, the evidence suggests that rTMS should be accompanied by motor training and rehabilitation, as this improves neuronal plasticity and the creation of long-lasting synapses. However, more research is required to determine the administration protocols for both techniques [16].

The presence or absence of post-stroke MEPs indicates that the corticospinal tract is functional and therefore an indicator of recovery [18]. Different studies on motor improvement, including a randomized controlled trial, have shown results where recovery was significantly higher in the groups that received TMS for five consecutive days compared to controls [19,20]. These groups showed motor improvement in clinical, neurophysiological, and imaging evaluations. For example, functional MRI studies have shown a positive post-intervention correlation in ipsilateral M1 and motor function, while the control groups have much lower motor excitability [19,20]. Although both LF-rTMS and HF-rTMS can be used to treat motor deficits after stroke, the use of low frequency is found to be safer and more effective in the recovery of motor function [3].

Upper limb

The use of LF-rTMS applied to the contralateral side of the lesion, specifically on M1 (motor cortex), is a useful tool in the recovery of the function of the upper limb. Most participants included in the clinical trials evaluating the efficacy of LF-rTMS were also receiving other types of motor training, a fact that must be taken into account when analyzing whether the effect of LF-rTMS is self-sufficient or necessitates other therapies [3].

Complex motor skills such as those performed by the hand and wrist improve significantly with LF-rTMS compared to the proximal part of the extremity. There is evidence that improvement in the upper limb motor function is more difficult than the recovery of lower limb activity [3]. This is mainly because the adaptive reorganization of the motor cortex after ischemic injury follows a pattern from the proximal to the distal limb, and studies have shown that the cortical representation of the hand also coordinates the movement of the proximal extremity through the muscles of the forearm, further limiting recovery [3]. Another theory refers to the complex movements of the hand being under the control of corticospinal projections, which, in turn, are the most affected after a stroke, and at the same time, these signals are easier to recover with rTMS, while non-complex activities are additionally controlled by brainstem projections which is not a target of rTMS [3]. Finally, regarding the short-term use of rTMS on the upper limb, some studies have shown mild but long-lasting improvement in activities such as grip strength and movement, with varying durations of rehabilitation ranging from 1-24 consecutive days [3].

Lower limb and balance

One of the consequences of stroke is the loss of balance and walking due to motor deficits in the lower limbs. The use of rTMS has shown some utility in the rehabilitation of such patients, as reported in a systematic review and meta-analysis reporting improvement in balance with a standard mean difference (SMD) of 0.38 (95% confidence interval (CI) = 0.07 to 0.069) and mobility with an SMD of -0.67 (95% CI = -1.08 to -0.26). The authors also suggested that rTMS might have longer-term benefits when compared to other methods; however, there was insufficient evidence to prove the effectiveness of TMS in lower limb rehabilitation [21]. Additionally, the use of rTMS, even for one day, has been related to better scores when compared to placebo in indices that evaluate motor impairment such as the Fugl-Meyer assessment [22]. When evaluating MEPs of the lower limbs, rTMS has demonstrated a higher level of improvement in chronic and non-chronic stroke patients and in patients who received the excitation mode of rTMS, but not in patients who received suppression [14,22].

It is important to note that rTMS therapy used on the lower limbs differs from the therapy of the upper limbs, as in about 90% of healthy individuals, the function of the upper extremity is controlled by the contralateral hemisphere. In comparison, while 70-80% of motor nerve fibers of the lower limbs originate in the contralateral hemisphere, the remaining 20-30% originate in the ipsilateral hemisphere; hence, the use of rTMS differs depending on the extremities being targeted [23]. Therefore, rTMS for the lower limbs can either target the hemisphere of the lesion (ipsilesional) or the contralateral one (contralesional). Studies with small samples and varying rTMS protocols have shown that there appears to be a slight benefit with ipsilesional stimulation; however, this claim should be further confirmed in larger samples with a standardized protocol and an experimental design [23].

Spasticity

Injury to cortical neurons decreases inhibitory inputs to fibers of the corticospinal tract, resulting in increased excitability of the spinal motor neurons and spasticity which can be targeted with rTMS [24]. An experimental study that applied five consecutive sessions (one session per day) of LF-rTMS on the affected motor cortex showed a reduction of spasticity symptoms in the lower limb, as assessed by the modified Ashworth scale (MAS). These results were maintained for a week after application, and electrophysiological changes were evident after 10 sessions [25].

When assessing its functionality in upper limb spasticity, a common complication after a stroke, Barros Galvão et al. combined physiotherapy with inhibitory rTMS (<1 Hz) for 10 consecutive days on the unaffected hemisphere which significantly reduced the MAS score for finger flexors and spasticity in the wrist [24]. In contrast, a systematic review and meta-analysis of randomized controlled trials reported no difference in the MAS score between rTMS and controls, but patients reported a better outcome in comparison to their status before therapy [26].

Aphasia

Approximately 30% (20-41%) of stroke survivors present with aphasia and only 5-10% report some type of recovery at one year of follow-up [27,28]. The use of rTMS in the right hemisphere of post-stroke patients has been shown to improve language functions. The way in which rTMS allows recovery from aphasia is based on its ability to stimulate neuroplasticity during the re-organization of language centers after a stroke [29].

There are multiple controversies regarding the function of the non-dominant hemisphere in post-stroke language recovery; however, rTMS, through both of its modalities (high frequency and low frequency), can stimulate the compensatory activity or inhibit inefficient nodes in the right hemisphere, increasing language recovery [29,30]. For instance, a double-blinded randomized trial that used LF-rTMS directed at the right pars triangularis (contralateral to the lesion side) for 10 consecutive days resulted in enhanced expression and comprehension in the rTMS group in comparison to the sham group (R^2^ > 0.7) [31].

Several studies show that TMS is more effective in motor aphasias and mixed aphasias with a motor predominance, for example, stimulation of Broca’s area with inhibitory frequencies has helped in improving the naming of objects or photos [1]. A systematic review of randomized controlled trials that included a total of 1,287 patients reported that LF-rTMS resulted in better recovery than sham rTMS and conventional rehabilitation procedures, especially in naming, language comprehension, and aphasia quotient. HF-rTMS was not associated with any improvement, and the study heterogeneity was high [30].

Dysphagia

Dysphagia is a common complication after stroke with an incidence in patients with hemispheric stroke ranging from 39% to 40% and in patients with mixed lesions ranging from 51% to 55% [32]. Furthermore, it increases the risk of complications such as aspiration and pneumonia (patients with dysphagia have a three-fold increased risk of developing pneumonia) [32]. Therefore, rehabilitation of patients with such disabilities is paramount to prevent complications and reduce morbidity and mortality.

Unlike other motor disorders, swallowing control has a bilateral representation in the cerebral cortex, which has led to significant heterogeneity in the results and protocols utilized in rTMS studies. This has precluded its use in dysphagia rehabilitation and its inclusion in the most recent rTMS evidence-based guideline [33]. However, a recent systematic review published in 2022 reported benefits in swallowing rehabilitation using both HF and LF-rTMS. Specifically, ipsilesional HF-rTMS results in an improvement in the standardized swallowing assessment (SSA) score immediately and at four weeks, and contralesional LF-rTMS results in immediate improvement in both the SSA and penetration aspiration scale (PAS) scores [34]. Furthermore, there appears to be a greater benefit in using rTMS for 10 consecutive days over direct transcranial current stimulation in post-stroke dysphagia [35]. Brainstem damage is the most common cause of post-stroke dysphagia. Stimulation of sensorimotor pathways has been associated with recovery; however, the exact mechanism through which rTMS results in recovery is still unknown, as published studies use multiple stimulation targets, duration, and frequencies [33-35].

Depression

Depression is a frequent sequela after stroke, with a pooled prevalence of 28%, 31%, 33%, and 25% at one month, six months, 12 months, and more than 12 months, respectively [36]. Moreover, post-stroke depression (PSD) has been associated with an increased risk of mortality with a hazard ratio of 1.59 (95% CI = 1.30 to 1.96) [37]. Despite its importance, rTMS guidelines have not included in their analysis either PSD or any neuropsychological impairment, but rather only analyzed the use of rTMS in depression by itself (i.e., not caused by stroke) giving HF-rTMS of the left dorsolateral prefrontal cortex with a figure-of-eight or H1 coil a grade of level A evidence [33].

The issue of the available evidence regarding PSD and rTMS is its low quality and high degree of heterogeneity [38]. However, there appears to be some benefit of rTMS in improving scores on the Hamilton depression rating scale, regardless of frequency (one day to two months) and stimulation site [39]. This might be the result of the aforementioned methodological deficiencies and heterogeneity. It would be illogical to assume that the frequency and the stimulation target have no effect on the rehabilitation results of PSD, especially when studies have shown that its presence depends on the location of the stroke; left hemisphere stroke, involving the dorsolateral prefrontal cortex or the dorsal anterior cingulated cortex, which coincidentally are part of the cortico-limbic pathways that play a central role in the regulation of emotions [40].

Current controversies and limitations

The absence of a standardized protocol for rTMS is a significant point of contention, as it hinders achieving optimal and effective therapeutic outcomes in the aftermath of a stroke [33,38]. The variability in the outcomes of rTMS therapy among patients may be attributed to factors such as genetic variations, the timing of rTMS application in relation to the acute or chronic phase post-stroke, the underlying pathology that led to the stroke, and the specific parameters employed during the administration of the therapy, including intensity, pattern, and duration.

According to the available literature, the efficacy of rTMS treatment for patients may be impacted by the existence of specific polymorphisms in genes responsible for serotonin carriers, serotonin receptors, and *BDNF *[2,4]. An example of a *BDNF *polymorphism is the rs6265 variant, which involves a substitution of valine with methionine at position 66 [41]. The genotypes Val/Met or Met/Met have been linked to a decreased neuroplasticity response and inferior therapeutic outcomes subsequent to rTMS therapy in comparison to Val/Val genotypes [41,42]. The genetic makeup of individuals who have suffered from stroke may play a crucial role in determining the effectiveness of therapy and warrants consideration.

The application of stimulation at the acute or chronic stage post-stroke is also a topic of controversy. A study showed that the utilization of LF-rTMS resulted in a noteworthy enhancement of motor function in stroke patients after a period of 45 days; however, the same study did not yield similar outcomes when the observation was conducted 90 days later [43]. Another study, in contrast, found that the application of brain stimulation has the potential to improve fine motor rehabilitation exclusively during the chronic phase following a stroke [44]. These might be explained due to the fact that outcomes exhibit variability based on the specific impairment and criteria employed to define and quantify them. Consequently, it is crucial to conduct an individualized analysis of the patient and develop a rTMS protocol that is most suitable for addressing their specific requirements. In brief, due to the diversity of research and outcomes currently present in the literature [45,46], it proves challenging to recommend a particular repetitive transcranial magnetic stimulation (rTMS) protocol.

Finally, randomized controlled trials focusing on rTMS often lack proper randomization techniques, the utilization of a proper control group (with sham stimulations), and often include a small number of participants. Perhaps, reproducibility of the protocols and heterogeneity of the results are due, in part, to the limited number of participants and the heterogeneity of outcomes used to measure success.

## Conclusions

Neurorehabilitation through rTMS is an evolving discipline that has resulted in strong recommendations by evidence-based guidelines regarding its use in post-stroke acute motor impairment, hand and wrist motor recovery, and chronic non-fluent aphasia. rTMS has shown benefits but with limited evidence and a high degree of protocol heterogeneity in the rehabilitation of lower limbs, spasticity, and dysphagia. PSD, although caused by dysregulations in the cortico-limbic pathways (similar to non-organic depression), has not been studied as thoroughly as non-organic depressive disorders which have shown great response to rTMS. More studies with methodological soundness and larger cohorts are required to assess the use of rTMS in all sequelae after a cerebrovascular event. The future of this technique seems bright, and multiple studies are showing the benefits of its use in reducing stroke morbidity.
